# Identification and Functional Characterization of a Geraniol Synthase UrGES from *Uncaria rhynchophylla*

**DOI:** 10.3390/plants14152273

**Published:** 2025-07-23

**Authors:** Xinghui Liu, Wenqiang Chen, Linxuan Li, Detian Mu, Iain W. Wilson, Xueshuang Huang, Yahui Xiang, Lina Zhu, Limei Pan, Deyou Qiu, Qi Tang

**Affiliations:** 1 National Research Center of Engineering Technology for Utilization of Botanical Functional Ingredients, College of Horticulture, Hunan Agricultural University, Changsha 410128, China; 18627362607@163.com (X.L.); 15958699677@163.com (W.C.);; 2 Guangxi Key Laboratory of High-Quality Formation and Utilization of Dao-di Herbs, National Center for TCM Inheritance and Innovation, Guangxi Botanical Garden of Medicinal Plants, Nanning 530023, China; 3 CSIRO Agriculture and Food, Canberra, ACT 2601, Australia; 4 Hunan Provincial Key Laboratory for Synthetic Biology of Traditional Chinese Medicine, Hunan University of Medicine, Huaihua 418000, China; 5 State Key Laboratory of Tree Genetics and Breeding, Research Institute of Forestry, Chinese Academy of Forestry, Beijing 100091, China

**Keywords:** terpene synthase, bioinformatics analysis, enzymatic assay, transient expression, terpenoid indole alkaloids

## Abstract

*Uncaria rhynchophylla*, a medicinal plant extensively used in traditional Chinese medicine, is an important plant source of terpenoid indole alkaloids (TIAs), but the mechanism of TIA biosynthesis at molecular level remains unclear. Geraniol synthase (GES) serves as a crucial enzyme in catalyzing the formation of geraniol from geranyl pyrophosphate (GPP) in various plants, but the functional characterization of the *GES* gene in *U. rhynchophylla* has not been investigated. In this study, a *GES* was identified and characterized through genome mining and bioinformatic analysis. Functional validation was performed via a protein catalysis experiment, transient expression in *Nicotiana benthamiana*, and methyl jasmonate (MeJA) induction experiments. The full-length *UrGES* gene was 1761 bp, encoding a protein product of 586 amino acids with an estimated 67.5 kDa molecular weight. Multiple sequence alignments and phylogenetic analysis placed UrGES within the terpene synthase g (TPS-g) subfamily, showing high similarity to known GESs from other plants. Enzymatic assays confirmed that recombinant UrGES catalyzed GPP conversion to a single product of geraniol. The transient expression of *UrGES* resulted in geraniol accumulation in *N. benthamiana*, further confirming its function in vivo. *UrGES* expression was observed in leaves, stems, and roots, where leaves had the highest transcript levels. Moreover, MeJA treatment significantly upregulated *UrGES* expression, which positively correlated with an increase in alkaloid content. This study functionally characterizes UrGES as a geraniol synthase in *U. rhynchophylla*, contributing to the current knowledge of the TIA biosynthetic pathway. These findings may offer insights for future metabolic engineering aiming to enhance TIA yields for pharmaceutical and industrial applications.

## 1. Introduction

Belonging to the Rubiaceae family, *Uncaria rhynchophylla* (Miq.) Miq. ex Havil. holds an important status in traditional Chinese medical practices and is primarily employed in the treatment of hypertension, convulsions, epilepsy, eclampsia, and encephalopathy [1]. The principal active constituents in *U. rhynchophylla* are terpenoid indole alkaloids (TIAs), including rhynchophylline, isorhynchophylline, corynoxeine, and isocorynoxeine [2], which have demonstrated therapeutic potential in managing Alzheimer’s disease, hypertension, and liver injury [3,4,5]. However, the current supply of these alkaloids is insufficient to meet medicinal demands due to their low natural abundance and the high cost of traditional cultivation methods. As such, synthetic biology and metabolic engineering strategies are promising alternatives, necessitating the mining of functional genes participating in TIA pathway.

Advances in high-throughput sequencing technologies have enabled genome and transcriptome sequencing of various medicinal plants, including alkaloid-rich species such as *Camptotheca acuminata* [6], *Ophiorrhiza pumila* [7], and *Dendrobium officinale* [8], thereby facilitating the identification of candidate biosynthetic genes and advancing our understanding of alkaloid secondary metabolite pathways. In *U. rhynchophylla*, several enzymes participating in TIA biosynthesis, along with key regulatory transcription factors, have been discovered. Through combined metabolomic and transcriptomic analyses, three key enzymes—strictosidine-β-D-glucosidase (UrSGD1), strictosidine synthase (UrSTR2), and L-tryptophan decarboxylase (UrTDC)—have been identified and functionally validated in vitro [9]. Additionally, the overexpression of *UrSTR1* and *UrSTR5* in transgenic hairy roots resulted in increased TIA accumulation, whereas RNA interference (RNAi)-mediated silencing of these genes led to a decline in TIA levels [10]. Under low-light conditions, the phytochrome-interacting factor UrPIF3 was shown to enhance the expression of *UrSGD* and *UrSTR*, whereas its silencing reduced isorhynchophylline content and significantly suppressed these genes [11]. Furthermore, the basic helix–loop–helix transcription factor UrbHLH1 has been observed to interact with promoters of 10-hydroxygeraniol dehydrogenase (*Ur10HGO*) and geraniol 10-hydroxylase (*UrG10H*), implicating a regulatory role in TIA biosynthesis [12].

Geranyl pyrophosphate (GPP) serves as an immediate precursor of monoterpenes and, catalyzed by terpene synthase (TPS), forms various monoterpene compounds [13]. GPP is produced by the condensation reaction of dimethylallyl diphosphate (DMAPP) and isopentenyl diphosphate (IPP), with these precursor molecules being synthesized via two distinct metabolic routes: the plastid-localized methylerythritol phosphate (MEP) pathway and the cytosolic mevalonate (MVA) pathway [14]. Accordingly, the classic TPS-based pathway in which geraniol synthase (GES) catalyzes GPP to produce geraniol in plastids [15] and a specific mechanism for the catalytic reaction of Nudix hydrolase in the cytoplasm [16]. The iridoid biosynthetic cascade commences with GPP conversion to geraniol; subsequently, secologanin is generated through a series of enzymatic reactions and condenses with indole pathway-derived tryptamine to form strictosidine, which is the direct precursor of TIAs such as vinblastine [15], camptothecin [17], and rhynchophylline [18]. As a TPS family member, GES is able to catalyze the conversion of GPP to geraniol [19]. *GES* genes were characterized across diverse plants, and encoded proteins were capable of catalyzing the geraniol formation in vitro [20,21,22,23]. In addition, a cytoplasmic-located Nudix hydrolase, RhNUDX1, was discovered in rose that catalyzes the conversion of GPP to geranyl monophosphate, which then further generates geraniol in the presence of a petal-derived phosphatase [24].

Although GES-mediated geraniol synthesis is a crucial step in the TIA biosynthesis pathway [18], no *GES* gene has yet been characterized in *U. rhynchophylla*. In this study, a *GES* gene was screened and cloned using genome data from *U. rhynchophylla* [25]. Bioinformatics analysis, including multiple sequence alignment and phylogenetic analysis, suggested its potential role in catalyzing geraniol formation from GPP. The catalytic function of UrGES was verified both in vitro (*Escherichia coli* BL21) and in vivo (*Nicotiana benthamiana*). We also assessed *UrGES* transcript levels in various *U. rhynchophylla* tissues (roots, stems, and leaves) and examined its response to methyl jasmonate (MeJA) treatment. This work offers preliminary evidence on the biosynthetic pathway of TIA in *U. rhynchophylla*, which may inform future investigations into metabolic engineering approaches for alkaloid production.

## 2. Results

### 2.1. Physicochemical Analysis of UrGES Protein

**Figure 1:**
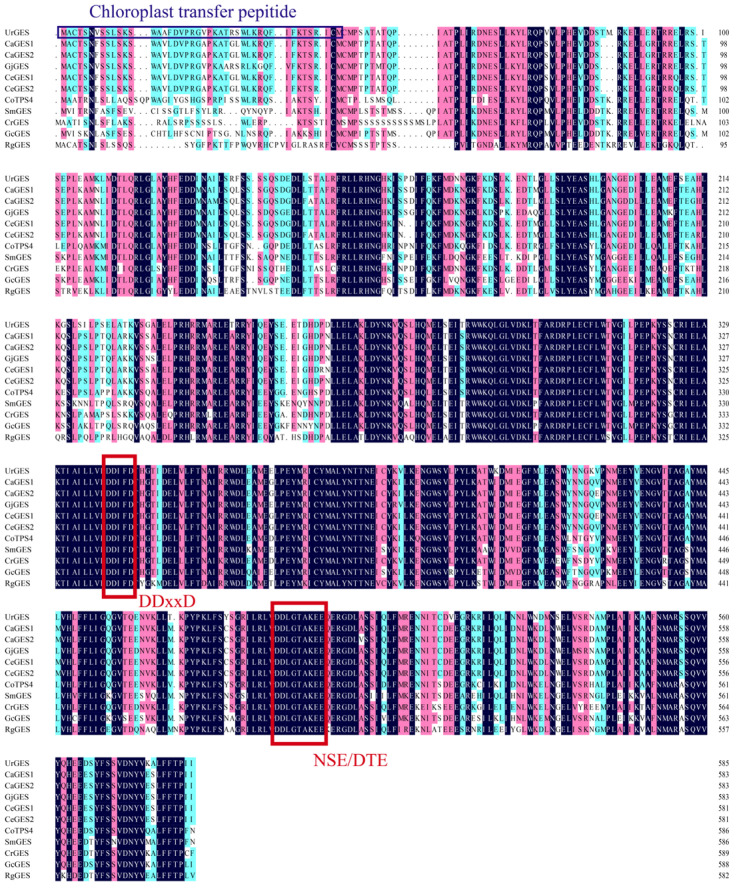
Amino acid sequence alignment of UrGES with geraniol synthases (GESs) in other plants. The highly conserved DDxxD and NSE/DTE motifs are highlighted within red frames. Chloroplast transfer peptide of UrGES is highlighted within the dark blue frame. The following protein sequences were used for multiple sequence alignment: CaGES1 (XP_027063605.1) and CaGES2 (XP_027063694.1) from *Coffea arabica*; GjGES (QCT83300.1) from *Gardenia jasminoides*; CeGES1 (XP_027169926.1) and CeGES2 (XP_027169969.1) from *Coffea eugenioides*; CoTPS4 (A0A7G5KLV3.1) from *Cananga odorata*; SmGES (QKS73430.1) from *Swertia mussotii*; CrGES (AFD64744.1) from *Catharanthus roseus*; GcGES (AJT35508.1) from *Gentiana crassa*; and RgGES (QJD13722.1) from *Rehmannia glutinosa*.

The *U. rhynchophylla* genome was assembled to a size of 627.72 Mb with a contig N50 of 1.80 Mb. Integrated genomic and transcriptomic analyses identified 46,909 genes [25], from which the coding sequence (CDS) of *UrGES* was obtained. The genome-derived *UrGES* sequence showed consistency with the full-length cloning results (Figure S1). Following validation, the *UrGES* sequence was registered in GenBank with accession number BankIt2972777 seq1 PV822539. The CDS comprised 1761 bp, translating into 586 amino acids, corresponding to an estimated molecular mass of 67.5 kDa. The theoretical pI, instability index and grand average of hydropathicity (GRAVY) were predicted to be 5.64, 51.14, and −0.266, respectively. Plant-mPloc predicted chloroplast localization for UrGES, with TargetP-2.0 identifying a 44-amino-acid chloroplast transit peptide at its N-terminus (Figure 1). Furthermore, transmembrane region analysis revealed no transmembrane domains in UrGES (Figure S2).

### 2.2. Identification and Phylogenetic Analysis of UrGES

**Figure 2:**
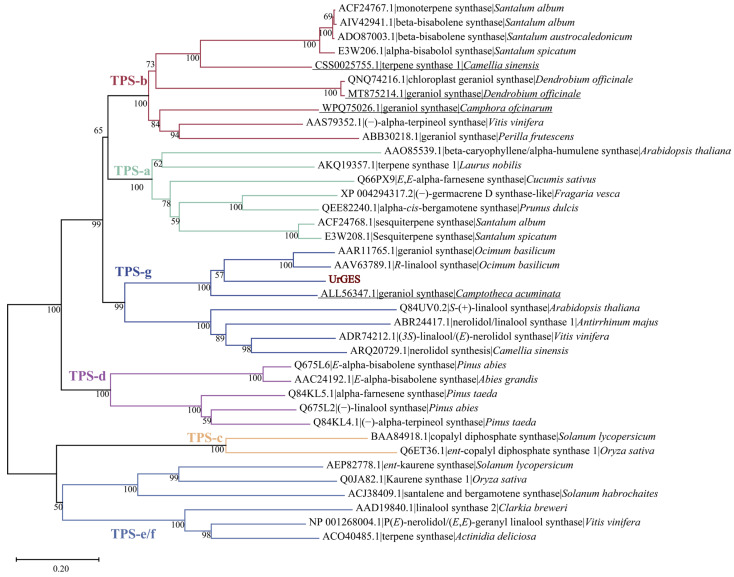
Phylogenetic comparison between UrGES and known terpene synthases (TPSs). MEGA 11 was applied to generate a neighbor-joining tree after aligning all full-length protein sequences with ClustalW. Red text was used to accentuate the presence of UrGES. The TPSs were categorized into seven subfamilies, with UrGES protein grouping within the TPS-g clade.

Ten GES sequences derived from various plants were chosen for comparison based on Basic Local Alignment Search Tool (BLAST) results on the National Center for Biotechnology Information (NCBI) platform. Comparative sequence alignment of UrGES with these closely related GESs has identified conserved DDxxD and NSE/DTE motifs. These motifs serve as characteristics of the TPS family [26] (Figure 1). TPS-g subfamily proteins are known to lack the RRX_8_W motif [27], and its absence in these sequences supports their classification within the TPS-g subfamily. To further explore the phylogenetic relationships between the UrGES protein and other TPSs in various subfamilies, a phylogenetic analysis was performed by the neighbor-joining algorithm. As shown in Figure 2, UrGES clustered within the TPS-g subfamily, which is typically found in angiosperms and participates in acyclic monoterpenes biosynthesis [27].

### 2.3. Heterologous Overexpression and Functional Characterization of Recombinant UrGES Protein

**Figure 3:**
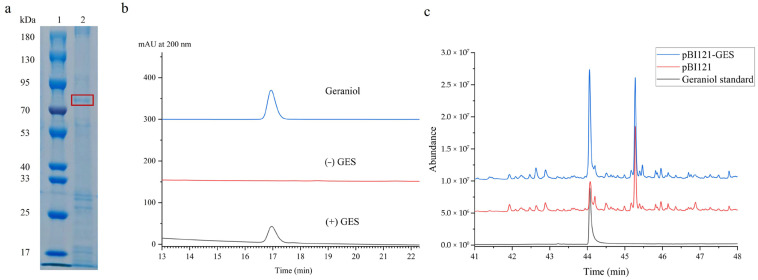
Detection of catalytic activity of UrGES protein in vitro and in vivo. (**a**) Sodium dodecyl sulfate–polyacrylamide gel electrophoresis (SDS-PAGE) of recombinant pET32a-UrGES protein. Lane 1 represents protein marker 10~180 kDa. Lane 2 indicates purified isopropyl-β-D-thiogalactoside (IPTG)-induced UrGES. The target recombinant protein UrGES is shown by the red box. (**b**) High-performance liquid chromatography (HPLC) analysis of UrGES catalytic activity. The blue line represents the geraniol standard; the red line, the product of incubating heat-inactivated recombinant UrGES protein with geranyl pyrophosphate (GPP); and the black line, the product of incubating recombinant UrGES protein with GPP. (**c**) Gas chromatography–mass spectrometry (GC-MS) detection of geraniol production in *Nicotiana benthamiana* leaves with *UrGES* transient overexpression. The black trace represents the geraniol standard, the red trace shows the extract from leaves transformed with the empty vector (pBI121) control, and the blue trace displays products from *UrGES*-expressing leaves.

For characterization of the catalytic function of the UrGES protein, the *UrGES* gene was inserted to the pET32a vector and expressed in a prokaryotic system, followed by Ni-NTA chromatography purification. The expression and purity of the pET32a-UrGES fusion protein were confirmed by sodium dodecyl sulfate–polyacrylamide gel electrophoresis (SDS-PAGE) analysis (Figure 3a), where the target band aligned with the expected molecular weight of the expressed protein. Upon induction with isopropyl-β-D-thiogalactoside (IPTG), pET32a-UrGES was expressed in the soluble fraction of the supernatant. The enzymatic capability of the purified protein was evaluated by employing GPP as the substrate. The high-performance liquid chromatography (HPLC) detection of reaction products showed that geraniol was produced in the UrGES-catalyzed reaction, with no detectable geraniol in the heat-inactivated control (Figure 3b). These results demonstrate that UrGES from *U. rhynchophylla* catalyzes the conversion of GPP to geraniol.

### 2.4. Functional Analysis of UrGES In Vivo

For further functional analysis of the UrGES protein in vivo, we performed *Agrobacterium*-mediated transient overexpression of *UrGES* driven by the CaMV 35S promoter in *N. benthamiana*. Polymerase chain reaction (PCR) analysis confirmed successful transgene expression, with *UrGES* transcripts being abundantly present in overexpression leaf sectors but undetectable in empty vector-transformed control leaves. Subsequent gas chromatography–mass spectrometry (GC-MS) analysis of *N. benthamiana* leaves revealed a notable increase in geraniol production in *UrGES*-overexpressing plants compared to controls (Figure 3c), consistent with the in vitro experimental results. These findings demonstrate that UrGES functions as a monofunctional enzyme responsible for transforming GPP into geraniol in plants.

### 2.5. Tissue Expression Pattern of UrGES

**Figure 4:**
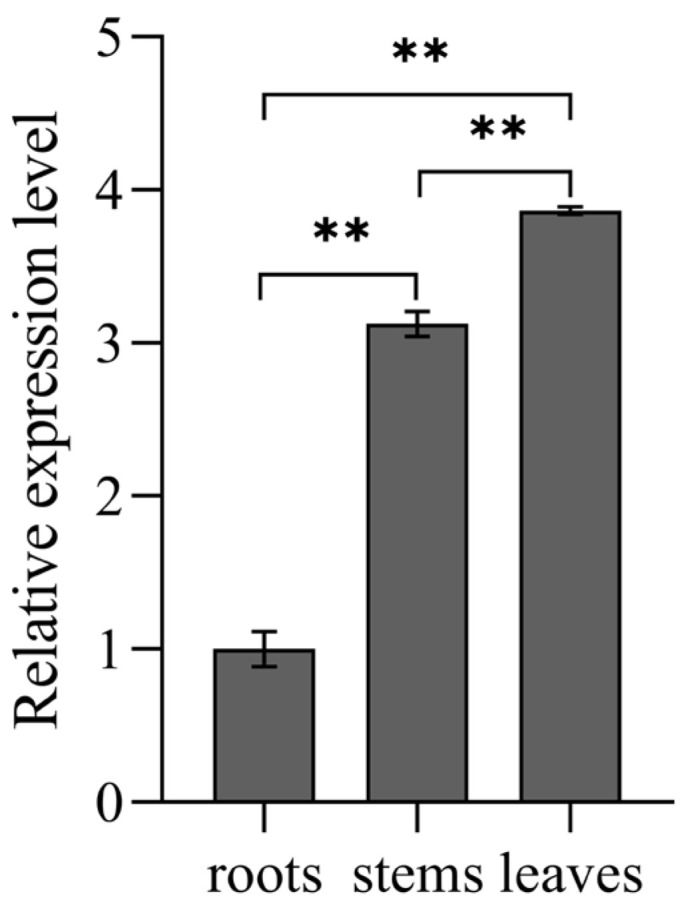
*UrGES* gene transcription patterns in different organs of *Uncaria rhynchophylla*. Values indicate means ± SD from triplicate assays. Double asterisks show a highly significant difference (*p* < 0.01) compared to the root.

Real-time quantitative PCR (qRT-PCR) was utilized for examining the *UrGES* expression degree across diverse tissues that comprised roots, stems, and leaves. As shown in Figure 4, *UrGES* was expressed in all detected tissues of *U. rhynchophylla*, and its relative expression level showed significant differences across different organs, encompassing roots, stems, and leaves. Leaf tissues showed the most abundant expression levels, with stems and roots exhibiting progressively lower expression. These outcomes indicate that leaves may function as the primary organ for *UrGES* transcription and thus potentially play a critical role in TIA precursor biosynthesis.

### 2.6. MeJA Promotes TIA Accumulation and UrGES Upregulation

**Figure 5:**
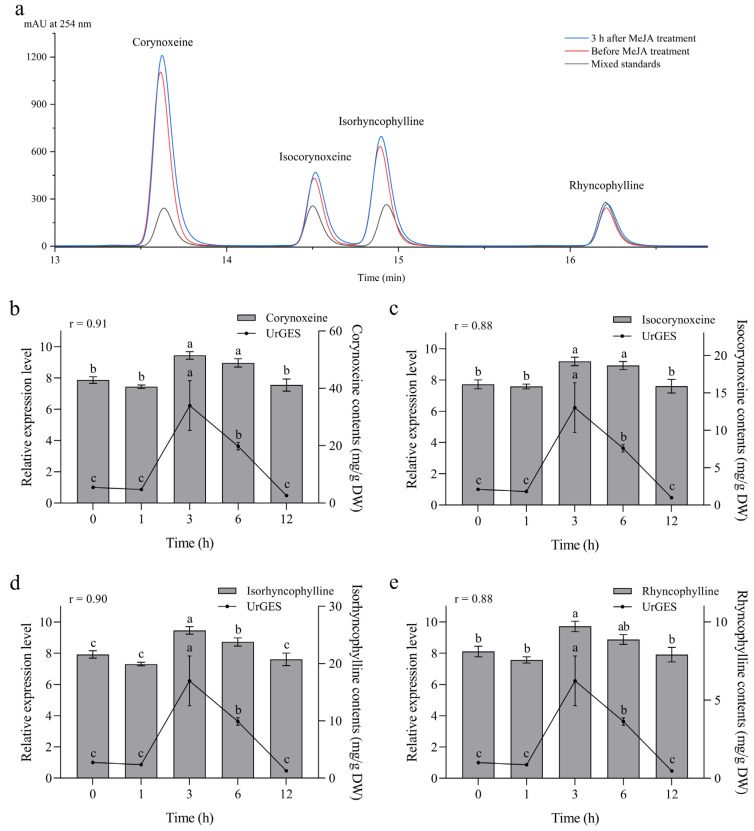
Upregulation of *UrGES* transcript levels and accumulation of terpenoid indole alkaloids (TIAs) in *U. rhynchophylla* in response to methyl jasmonate (MeJA). (**a**) HPLC chromatograms of standards and samples treated at different time points. The black line indicates mixed standards, including rhynchophylline, isorhynchophylline, corynoxeine, and isocorynoxeine; the red line denotes the control not treated with MeJA, and the blue line denotes the leaf samples treated with MeJA for 3 h. (**b**–**e**) Correlation analysis between the *UrGES* transcript levels and the concentrations of the four TIAs of *U. rhynchophylla* at different treatment times. Data indicate the mean ± standard deviation from three repeats. The presence of distinct lowercase letters indicates statistical significance (*p* < 0.05), and *r* stands for the Pearson correlation coefficient.

MeJA is an endogenous phytohormone with multiple physiological functions and is crucial for regulating the accumulation of plant secondary metabolites [28]. To investigate whether exogenous MeJA affects alkaloid accumulation in *U. rhynchophylla*, we quantified the contents of rhynchophylline, isorhynchophylline, corynoxeine, and isocorynoxeine in leaves at different treatment times using HPLC. The chromatographic peaks of the leaf samples and standards are shown in Figure 5a, and MeJA treatments induced the accumulation of all four TIAs. Compared with the control at 0 h, the content of all TIAs did not change significantly at 1 h, peaked at 3 h, and subsequently declined progressively to approach the near-original values after 12 h (Figure 5). To investigate whether the *UrGES* gene responded to MeJA induction, we analyzed its relative expression levels by qRT-PCR. Similar to TIA accumulation, *UrGES* transcript levels peaked at 3 h and then declined slowly, returning to near-original levels, suggesting that MeJA effectively upregulated *UrGES* expression. In addition, correlation analysis showed a significant positive relationship between *UrGES* expression pattern and the content changes in these four TIAs (Pearson correlation coefficient *r* > 0.8, *p* < 0.01) (Figure 5). Taken together, these results suggest that in *U. rhynchophylla* plants, *UrGES* is likely to contribute to TIA biosynthesis by catalyzing the formation of intermediates, a process regulated by MeJA signaling transduction.

## 3. Discussion

*U*. *rhynchophylla*, an important medicinal plant resource in China, is rich in TIAs, which exhibit significant pharmacological activities, particularly in the treatment of hypertension and neurological disorders [29,30,31]. To investigate the biosynthetic mechanisms underlying TIA production in *U. rhynchophylla*, genome sequencing of this species has recently been completed [25]. Elucidating the biosynthetic pathways and their regulatory mechanisms is essential for enhancing TIA accumulation and remains a key research focus [32,33,34].

In this study, a *GES* gene was identified based on the genome sequencing results of *U. rhynchophylla*. Sequencing verification confirmed the successful cloning of a 1761 bp *UrGES* gene encoding 586 amino acids. Bioinformatic characterization using the ExPASy website revealed that UrGES exhibits characteristics of a weakly acidic (pI 5.64) and unstable (instability index 51.14) protein. With a GRAVY value of −0.266 and transmembrane region analysis showing no transmembrane domains, these results collectively suggested that UrGES is a hydrophilic protein. In plants, geraniol is known to be synthesized through two spatially separated pathways: the common pathway, which uses GPP produced by the MEP pathway as a substrate and is catalyzed by GES proteins in the plastid; and the atypical route, which involves cytoplasmic NUDX proteins converting GPP to geraniol [15,16]. UrGES was predicted to localize to the chloroplast, along with the presence of a chloroplast transit peptide at its N-terminus, as determined using Plant-mPLoc and TargetP-2.0. These predictions aligned with the verified chloroplast targeting of GES proteins across various plant species, such as CoTPS4 (*Cananga odorata*) [20], CsTPS10 (*Camellia sinensis*) [35], PbTPS5, and PbTPS9 (*Phalaenopsis bellina*) [36]. These findings suggest that the GES protein in *U. rhynchophylla* likely functions in the plastid, using MEP-derived GPP as a substrate for monoterpene biosynthesis via the TPS-based pathway.

During early land plant evolution, two duplications of the ancestral TPS led to the emergence of three major TPS clades, now classified as TPS-c, TPS-e/f, and the TPS-h/d/a/b/g subfamily [37]. Among these, the TPS-a, TPS-b, and TPS-g subfamilies, existing predominantly in angiosperms, play specialized roles in terpene biosynthesis [37,38]. The TPS-a subfamily proteins are involved in the synthesis of sesquiterpenes and diterpenes in monocotyledonous and dicotyledonous plants [39]. In contrast, the closely related TPS-b and TPS-g mainly take part in the synthesis of monoterpene, whereas TPS-b contains a RRX8W motif at N-terminus, which is a crucial domain for divalent metal ion binding and is absent in TPS-g [40]. Previous studies have shown that GES proteins from different plants have been classified into the TPS-b or TPS-g subgroup [35,36,41] (Figure 2). Amino acid sequence analysis revealed that UrGES possesses typical structural features of the TPS-g subfamily. Phylogenetic analysis further classified UrGES into a TPS-g subfamily that primarily consists of acyclic terpene synthases. Four experimentally validated GES enzymes from other plant families (highlighted with underlining in Figure 2) were included in our phylogenetic analysis [19,21,23,42]. The resulting tree revealed their distribution across distinct clades of the TPS family (TPS-b and TPS-g), and despite their substantial sequence divergence, all retained the essential NSE/DTE and DDxxD conserved motifs (Figure S3). This result suggests the occurrence of convergent evolution, where distinct TPS subfamilies independently evolved the same function and evolutionary divergence from different plant families [43]. These bioinformatics-derived characteristics not only suggested UrGES to be a typical member of the TPS family, but also provided testable hypotheses for its enzymatic function in plastidial monoterpene biosynthesis. Geraniol was identified as the major product of the recombinant UrGES protein enzymatic assays in vitro, in agreement with previous studies. For example, CoTPS4 in *C. odorata* belongs to TPS-g and exclusively produces geraniol from GPP [20]. To provide in vivo evidence of UrGES function, the gene was heterologously expressed in tobacco. Figure 3c shows that *UrGES*-overexpressing transgenic plants produced relatively high levels of geraniol compared to the controls. These results suggest that UrGES functions specifically as a geraniol synthase. Similarly, transient *CoGES* (*Camphora officinarum*) expression in *N. benthamiana* resulted in increased geraniol accumulation (relative content), whereas the empty vector control only produced basal levels of geraniol [23]. Functional characterization revealed that CsTPS10 from tea plant (*C*. *sinensis*) failed to produce detectable metabolites in a prokaryotic expression system, whereas transient expression in *N. benthamiana* leaves yielded measurable geraniol [35]. The ectopic expression of *LiTPS2* from *Lilium* ‘Siberia’ in tobacco significantly increased monoterpene levels in both flowers and leaves, with linalool content in transgenic flowers exceeding that of wild-type plants 2–3-fold [44]. Studies have validated geraniol synthase’s function in tea plants, showing that silencing *CsTPS1-AS* reduces geraniol accumulation, increases pathogen susceptibility, and downregulates defense-related and salicylic acid pathway genes, whereas silencing *CsTPS1* has no significant effect, indicating its role in geraniol formation and defense through alternative splicing [19]. Given these findings, the functional characterization of UrGES in its native host is essential, particularly to validate its proposed role as the key initiating enzyme in the iridoid pathway.

MeJA is involved in responses to various stresses, as well as regulating secondary metabolite accumulation, including terpenes and alkaloids [45,46]. Numerous genes and transcriptional regulators associated with the TIA pathway in *Catharanthus. roseus* have been shown to respond to MeJA induction [46]. The MeJA-responsive transcription factor ORCA3 enhances biosynthesis of TIA by upregulating metabolic genes in *C. roseus*, highlighting a connection between the responses of jasmonic-acid-mediated stress and plant secondary metabolite production [47]. Moreover, MeJA treatment induced significant upregulation of the *CrGES* transcription level in *C. roseus* cells [15]. In MeJA-treated *Rauvolfia verticillata* hairy roots, the *RvTDC* expression was slightly upregulated, which was positively correlated with ajmalicine accumulation [48]. Similarly, MeJA-induced TIA accumulation and the upregulation of *UrGES* gene expression were observed in *U. rhynchophylla* leaves (Figure 5), suggesting that the biosynthesis of TIA in *U. rhynchophylla* is regulated by MeJA signaling and that *UrGES* is an elicitor-responsive gene activated by MeJA induction. Furthermore, strong positive correlation between *UrGES* expression and MeJA-induced TIA accumulation implies that UrGES might play a key role in TIA biosynthesis. However, the precise molecular mechanisms through which MeJA signaling regulates TIA biosynthesis in *U. rhynchophylla* remain unclear, warranting further investigation.

## 4. Materials and Methods

### 4.1. Plant Materials and Growth Conditions

The plant materials of *U. rhynchophylla* were originally collected from the Guangxi Botanical Garden of Medicinal Plants (Nanning City, Guangxi Province, China; 108.19° E, 22.49° N) and identified by Prof. Shugen Wei. All *U. rhynchophylla* materials used in this study were tissue-cultured plantlets obtained through multiple subculture generations from these original materials, and the specimen is currently deposited at the Yuelushan Laboratory (Room 121, Building 2, Changsha, China) with the voucher number GT20250115. The tissue-cultured seedlings were grown on Murashige and Skoog (MS) Base Salts with vitamins (Coolaber, Beijing, China) [49], supplemented with 25 g/L sucrose, 10 g/L agar, 0.2 mg/L 1-naphthaleneacetic acid (NAA), and 1 mg/L 6-benzylaminopurine (6-BA). The cultures were maintained in a growth chamber with a 12 h photoperiod (12 h light/12 h dark), 140 µmol/m^2^/s^1^ light intensity, and 60% humidity at 25 °C. These plant tissue culture conditions were modified from established protocols [50,51]. Two-month-old seedlings grown in tissue culture were sampled to obtain roots, stems, and leaves and snap-frozen using liquid nitrogen, before long-term preservation at −80 °C for subsequent total RNA extraction. Five-month-old *N*. *benthamiana* plants were cultivated under controlled environmental conditions (16 h light and 8 h dark cycles) in biotron and subsequently used for transient expression assays.

### 4.2. Total RNA Extraction and cDNA Synthesis

The FastPure Cell/Tissue Total RNA Isolation Kit (Vazyme, Shanghai, China) was employed for extraction of total RNA from various *U. rhynchophylla* organs, such as roots, stems, and leaves, strictly following the guidelines provided by the manufacturer. The Micro Drop instrument (BIO-DL, Shanghai, China) was applied for the calculation of the concentration and purity of the obtained RNA. Electrophoresis was conducted using 1% agarose gels to evaluate the RNA integrity. After that, 500 ng of total RNA underwent reverse transcription utilizing the Evo M-MLV RT Mix Kit (Accurate Biology, Changsha, China). The cDNA obtained served as template for qRT-PCR analysis. A PrimeScript ™ II 1st Strand cDNA Synthesis Kit (TAKARA, Dalian, China) was utilized to produce the cDNA needed for the RT-PCR that followed.

### 4.3. Molecular Cloning and Plasmid Construction for UrGES

The CDS of *UrGES* was derived from the *U. rhynchophylla* genome database. To clone the full-length *UrGES* gene, sequence-specific primers were designed by Snapgene 6.0.2 software and commercially synthesized. The PCR experiment was carried out utilizing cDNA as template with PrimeSTAR (Takara, Dalian, China) on an Applied Biosystems Veriti 96-well Thermal Cycler (Thermo Fisher Scientific, Waltham, MA, USA). The cycling condition were as follows: 98 °C for 3 min, 35 cycles of 98 °C for 10 s, 55 °C for 15 s, and 72 °C for 1.5 min, followed by 72 °C for 5 min. The PCR-amplified fragments were purified with a GeneJET Gel Extraction Kit (Thermo Fisher Scientific, Waltham, MA, USA) and subsequently ligated into pMD^TM^19-T plasmid. For prokaryotic expression, the full-length *UrGES* was inserted into the pET32a plasmid that had been linearized by *EcoR* I and *BamH* I restriction enzymes. In order to build an overexpression vector, the CDS of *UrGES*, from which the stop codon had been removed, was ligated into the pBI121 plasmid linearized with *BamH* I. *BamH* I-digested pBI121 plasmid. The transformation of recombinant plasmids pET32a-*UrGES* and pBI121-*UrGES* into the *E. coli* DH5α was conducted, and Shanghai Sangon performed the sequencing work. The extraction of recombinant vectors was performed using the SanPrep Column Plasmid Mini-Preps Kit (Sangon, Shanghai, China) following the manufacturer’s instructions. This study utilized the primer sequences provided in Table 1.

### 4.4. Bioinformatics Analysis of UrGES Protein

An online website ExPASy (https://web.expasy.org/protparam/ (accessed on 20 January 2025)) was employed for computing the amino acid number, molecular weight, theoretical pI, and instability index as well as GRAVY. Furthermore, the Plant-mPLoc website (http://www.csbio.sjtu.edu.cn/bioinf/plant-multi/ (accessed on 20 January 2025)) and TargetP-2.0 tool (https://services.healthtech.dtu.dk/services/TargetP-2.0/ (accessed on 13 March 2025)) were utilized to predict the subcellular localization of UrGES. The TMHMM website (https://dtu.biolib.com/DeepTMHMM (accessed on 20 January 2025)) was used for the prediction of transmembrane topology. TPS amino acid sequences from various plants are available from NCBI (https://www.ncbi.nlm.nih.gov/ (accessed on 7 January 2025)). The multiple sequence alignments were conducted by employing DNAMAN 7 software. Using the neighbor-joining approach along with 1000-replicate bootstraps, the phylogenetic analysis was conducted using MEGA 11 software.

### 4.5. Heterologous Overexpression and Purification of UrGES

After sequence confirmation, the validated pET32a-*UrGES* vector was introduced into *E. coli* BL21(DE3) and the monoclonal strain was cultivated in Luria–Bertani broth containing Ampicillin at 37 °C with 200 rpm shaking overnight. To stimulate *UrGES* expression, IPTG was supplemented to achieve 0.1 mM final concentration when the OD_600_ of the bacterial culture reached 0.6–0.8. Following a 20 h incubation period at 16 °C and 140 rpm, the cell pellets were harvested through centrifugation (5000 rpm, 10 min). After that, cells were resuspended with Binding/Wash Buffer (Sangon, Shanghai, China) and lysed through 20 min ice-bathed sonication (SCIENTZ-IID Ultrasonic Cell Disruptor, Scientz, Ningbo, China). The soluble fraction was obtained via centrifugation at 10,000 rpm, 4 °C, 30 min. Protein purification was then performed using affinity chromatography. Following the protocol, the supernatant was put into an equilibrated gravity column containing Ni-NTA 6FF Sepharose (Sangon, Shanghai, China). The proteins were further concentrated using an ultrafiltration tube at 4000 rpm, 4 °C. Finally, the detection of purified proteins was carried out using SDS-PAGE, and their concentration was determined using a Micro Drop (BIO-DL, Shanghai, China).

### 4.6. Catalytic Activity Assay of UrGES Protein

To measure the enzyme activity of the recombinant UrGES protein, an assay was conducted using a reaction system of 200 µL phosphate-buffered saline (PBS, pH 7.5). This mixture contained the UrGES protein along with 1 mM dithiothreitol (DTT), 1 mM MgCl_2_, and 0.1 mM MnCl_2_. Subsequently, the substrate GPP was added to initiate the enzymatic assay, which was carried out in three repetitions. High-temperature inactivated proteins of equal mass were used as a control. The incubation of the reaction solution was conducted at 37 °C for 2 h. Subsequently, the reactions were terminated through the addition of an equal volume of methanol.

To detect geraniol, the obtained samples were passed through 0.22 μm membrane filters, and then analyzed using HPLC (Thermo Scientific™ UltiMate™ 3000 High Performance Liquid Chromatography System, Thermo Scientific, Waltham, MA, USA) equipped with a GL-Sciences GL-C18 column (250 mm × 4.6 mm, 5 μm, GL Sciences, Tokyo, Japan). The sample injection volume was 10 μL, and the mobile phase was an isocratic mixture of water and acetonitrile in a 43:57 ratio. The system operated at a flow rate of 1 mL/min with the chromatographic column fixed at 30 °C, while UV absorption occurred at 200 nm [21].

### 4.7. Transient Heterologous Expression Assay of UrGES in N. benthamiana Leaves

After sequencing validation, the recombinant pBI121-*UrGES* vector along with the control pBI121 was independently introduced into *A. tumefaciens* GV3101 strain. Transient expression assays in *N. benthamiana* were carried out by infiltrating *A. tumefaciens* cultures (OD_600_ = 0.6) into tobacco leaves. To minimize variability, the bacterial suspensions of the control vector and pBI121-*UrGES* were co-infiltrated into different sites of the same leaf. Following infiltration, the tobacco was placed under dark conditions for 12 h, followed by a light/dark 16 h/8 h cycle for 2 days at 24 °C. The infiltrated leaf regions were then excised, snap-frozen using liquid N_2_, and then preserved in a −80 °C freezer for subsequent studies.

For geraniol detection, 500 mg leaf tissue samples were weighed in 30 mL headspace vials and equilibrated for 30 min at ambient temperature. Volatile compounds were adsorbed at 60 °C for 60 min using a 50/30 μm DVB/CAR/PDMS SPME fiber (Supelco, Bellefonte, PA, USA). GC-MS analysis was performed using an Agilent 7890B-7000C triple quadrupole system (Agilent, Santa Clara, CA, USA) fitted with a DB-WAX IU capillary column (30 m × 0.25 mm × 0.25 μm; Agilent, Santa Clara, CA, USA), using the method previously described [52].

### 4.8. Expression Pattern of UrGES Gene

Fluorescent quantitative primers generated via Beacon Designer 7.0 can be seen in Table 1. Using an ABI 7300 Real-Time PCR System (Thermo Fisher Scientific, Waltham, MA, USA), the qRT-PCR was performed in an 8-Strip Tube. The 20 μL reaction system was prepared as follows: 2 μL cDNA, 10 μL 2X SYBR Green Pro Taq HS Premix, 0.4 μL ROX Reference Dye (both from Accurate Biology, Changsha, China), and 0.4 μL of each primer (sense and antisense). The final volume was adjusted to 20 μL using RNase-free water. Prior to usage, the template cDNA was diluted ten times. The processes involved in the PCR amplification reaction were as follows: 95 °C for 3 min, followed by 40 cycles of 95 °C for 15 s and 60 °C for 30 s, terminating with a dissociation stage. The relative quantification of the *UrGES* gene was determined by applying the 2^−∆∆CT^ approach, where *UrSAM* served as a housekeeping gene. GraphPad Prism 8.0 software was employed for visualizing the relative expression profile of *UrGES* and analyzing the significant differences.

### 4.9. Analysis of U. rhynchophylla Alkaloid Content After MeJA Treatment

Two-month-old tissue-cultured plantlets of *U. rhynchophylla* with similar growth status were selected for MeJA treatments. The foliar application was performed by evenly spraying 100 μM MeJA solution until runoff. Untreated (0 h) samples served as controls, and leaf samples were collected at 1, 3, 6, and 12 h post-treatment. Two portions of the samples were separated: a portion was utilized for RNA extraction, while the other was used to analyze *U. rhynchophylla* alkaloid accumulation (rhynchophylline, isorhynchophylline, corynoxeine, and isocorynoxeine). Each group underwent three biological replicate experiments.

The leaves of *U. rhynchophylla* were dried at 60 °C, finely ground, and sieved using a 60-micron mesh before collection. Each sample was measured at 0.1 g before the alkaloids were extracted using 1 mL of 80% aqueous methanol. The resulting homogenate underwent sonication (SB-3200DT, Scientz, Ningbo, China) for one hour, and then centrifuged at a speed of 12,000 rpm for five minutes. Following centrifugation, the supernatants were filtered through a 0.22 μm nitrocellulose filter (Jinteng, Tianjin, China). Reference standards, including rhynchophylline, isorhynchophylline, corynoxeine, and isocorynoxeine (each 1 mg), were precisely weighed and then dissolved in 1 mL of dimethyl sulfoxide (DMSO). A standard curve was constructed using seven-point serial dilutions prepared from the stock solution. Quantification of these four TIAs was conducted using HPLC in accordance with an established method from prior research [18].

## 5. Conclusions

This study identified and functionally characterized a novel geraniol synthase gene (*UrGES*) in the *U. rhynchophylla* genome. Multiple sequence alignment and phylogenetic analysis classified UrGES within the TPS-g subfamily. The enzymatic function was validated through both in vitro catalytic assays and transient expression in *N. benthamiana*. Furthermore, *UrGES* was observed to be transcribed the most in leaves, with significant transcriptional induction by exogenous MeJA treatment correlating with increased accumulation of TIAs. Collectively, this study indicates that UrGES serves as a pivotal enzyme in the TIA biosynthetic pathway and plays a potential role in TIA accumulation. Furthermore, the identified *UrGES* gene resource offers a potential new metabolic engineering approach to enhance iridoid and alkaloid biosynthesis in future studies.

## Figures and Tables

**Table 1:** List of primers.

Primer Name	Sequences (5′ to 3′)	Usage
*UrGES*-F	ATGGCTTGCACAAGTAACGT	Gene cloning
*UrGES*-R	TCAAGTAATTATAGGAGTGAAAAA	
pET32a-*UrGES*-F	GCCATGGCTGATATCGGATCCATGGCTTGCACAAGTAACG	Prokaryotic expression
pET32a-*UrGES*-R	TTGTCGACGGAGCTCGAATTCTCAAGTAATTATAGGAGTGAAAAACAGAG	
pBI121-*UrGES*-F	ACGGGGGACTCTAGAGGATCCATGGCTTGCACAAGTAACG	Transient expression
pBI121-*UrGES*-R	GGACTGACCACCCGGGGATCCAGTAATTATAGGAGTGAAAAACAGAGC	
*UrGES*-qRT-F	AGGTCCAATCACTTCATCA	qRT-PCR
*UrGES*-qRT-R	GCATTCAAGCGGTCTATC	

## Data Availability

The primary data generated during this research is available within the published article and its Supplementary Materials.

## Supplementary Materials

The following supporting information can be downloaded at https://www.mdpi.com/article/10.3390/plants14152273/s1: Figure S1: Total RNA extraction and *UrGES* gene cloning; Figure S2: Prediction of the transmembrane structural domains of UrGES protein using TMHMM website; Figure S3: Amino acid sequence alignment of UrGES with verified GESs in other plants; Figure S4: HPLC standard curve of TIAs.
