# Supplementary material for: Identification and Functional Characterization of a Geraniol Synthase UrGES from Uncaria rhynchophylla
Source: Plants (Basel). 2025 Jul 23;14(15):2273. doi: 10.3390/plants14152273 (PMC12348840; doi:10.3390/plants14152273)
Supplement: Supplementary file 1 [file plants-14-02273-s001.zip › plants-3749411-supplementary.pdf]

Supplementary Material

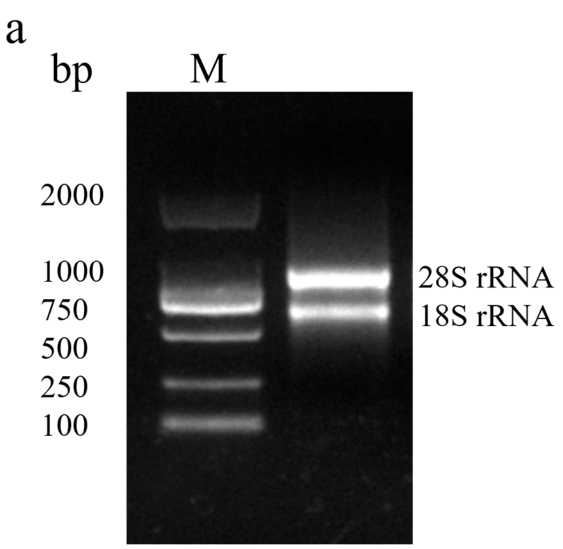

Figure S1. Total RNA extraction and *UrGES* gene cloning. (a) Total RNA from *Uncaria rhynchophylla*. (b) Polymerase chain reaction (PCR) products of the *UrGES* gene.

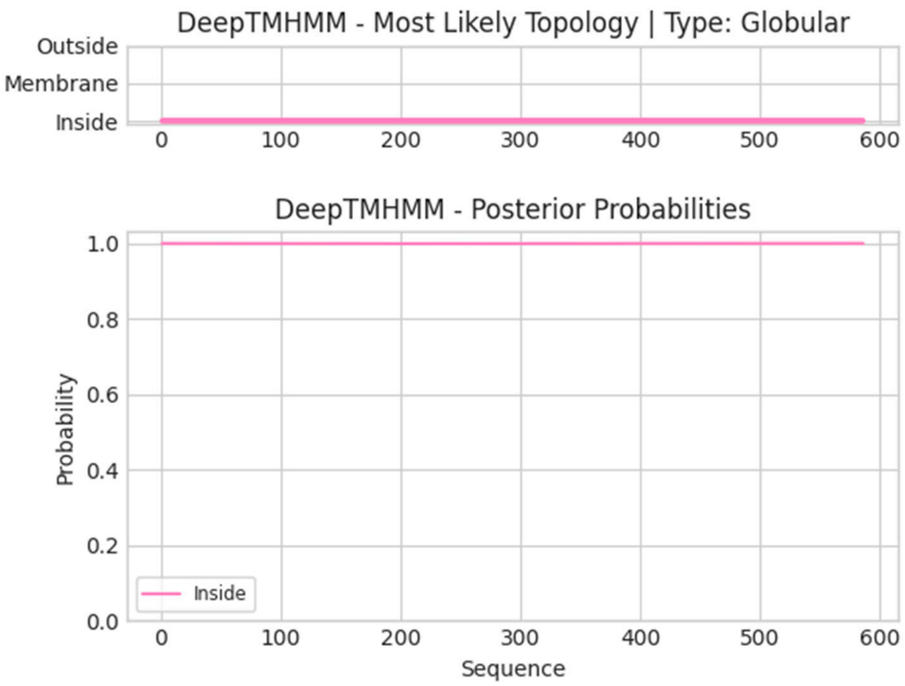

Figure S2. Prediction of the transmembrane structural domains of UrGES protein using TMHMM website.

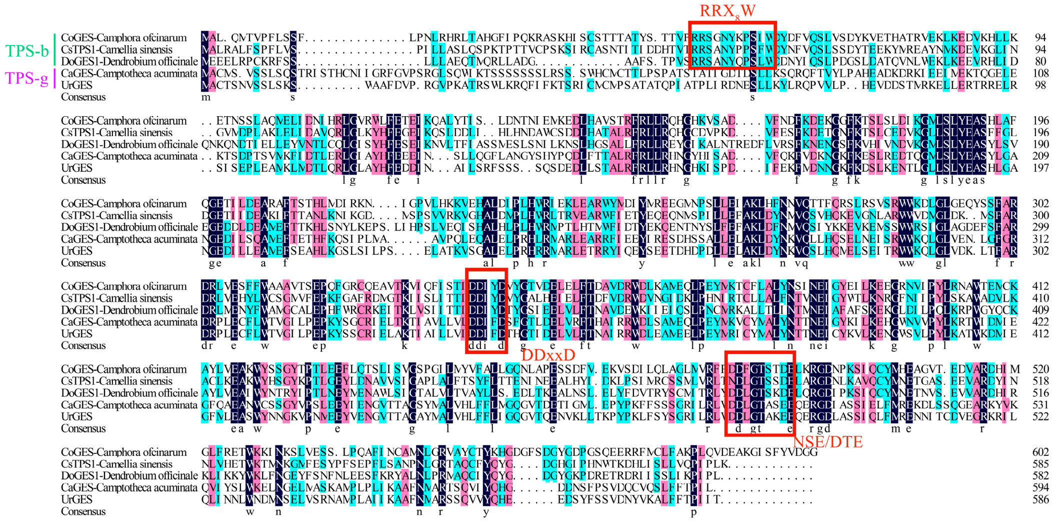

Figure S3. Amino acid sequences alignment of UrGES with verified GESs in other plants. The highly conserved motifs are highlighted within red frames.

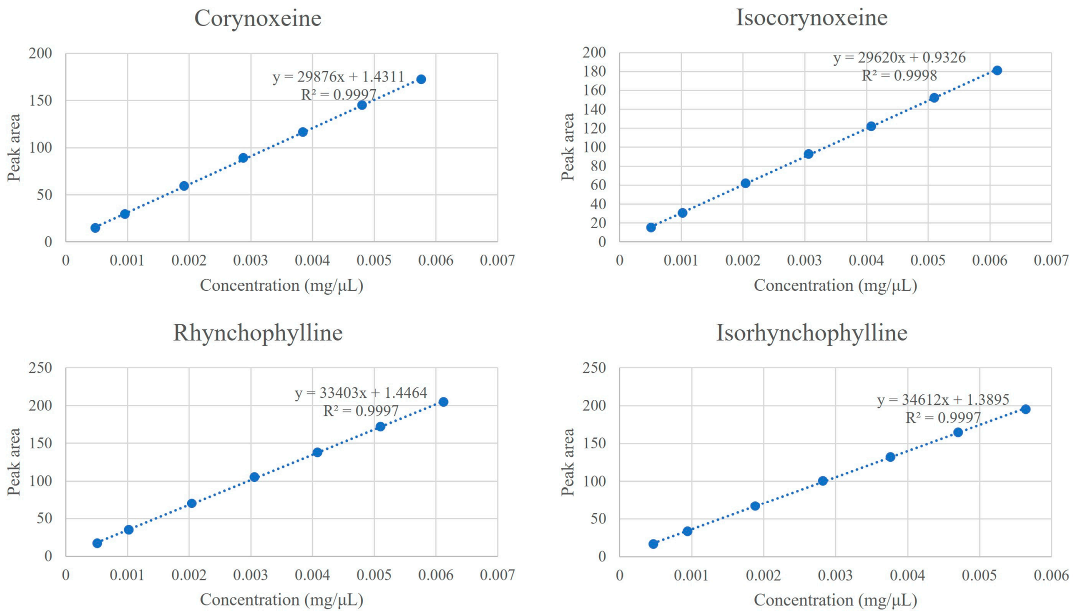

Figure S4. HPLC standard curve of TIAs.
